# Eosinophilic Fasciitis in a Young Adult: A Rare Presentation Potentially Triggered by the Epstein-Barr Virus

**DOI:** 10.7759/cureus.96207

**Published:** 2025-11-06

**Authors:** Bsher Almaalouli, Harini Reddy Choula, Maya Khasho, Hesham Nasser

**Affiliations:** 1 Internal Medicine, University of Central Florida College of Medicine, Orlando, USA; 2 Internal Medicine, Hospital Corporation of America (HCA) Florida North Florida Hospital, Gainesville, USA

**Keywords:** connective tissue disorder, corticosteroid therapy, eosinophilic fasciitis, epstein–barr virus, iron deficiency anemia, splenomegaly

## Abstract

Eosinophilic fasciitis (EF) is a rare fibroinflammatory disorder that may mimic systemic autoimmune disease or hematologic malignancy. We report the case of a 19-year-old male patient who presented with progressive swelling, stiffness, and burning pain of the extremities. Laboratory studies were significant for inflammatory marker elevation and polyclonal hypergammaglobulinemia. Epstein-Barr virus (EBV) testing demonstrated a positive polymerase chain reaction (PCR) test and IgG with negative IgM, suggesting prior infection with possible viral reactivation. Imaging showed splenomegaly and diffuse fascial edema, and a deep fascial biopsy confirmed EF. The patient was treated with high-dose corticosteroids with rapid clinical improvement. This case highlights the diagnostic complexity of EF and raises the possibility of EBV as an immunologic trigger.

## Introduction

Eosinophilic fasciitis (EF) is a rare immune-mediated connective tissue disorder first described by Shulman in 1974 [1]. It primarily affects the fascia, the thin layer of connective tissue that surrounds muscles, leading to painful swelling, skin tightening, and limited joint mobility. Because these findings may resemble systemic sclerosis, EF is often challenging to recognize clinically [2,3]. The exact cause of EF remains unclear, but most cases are considered idiopathic. Reported triggers include strenuous exercise, trauma, infections, and, less commonly, malignancies [2,4,5]. Among infectious triggers, Epstein-Barr virus (EBV) has been associated with immune dysregulation and chronic inflammation in several autoimmune diseases, suggesting it may play a role in EF pathogenesis [6]. We present a case of EF in a young male patient with systemic features, including anemia and splenomegaly, raising the question of EBV as a possible contributing factor.

## Case presentation

A 19-year-old previously healthy male patient presented with a one-month history of progressive swelling, stiffness, and burning discomfort in his lower extremities, later involving the forearms. He reported increasing fatigue but denied fever, night sweats, or weight loss.

**Figure 1:**
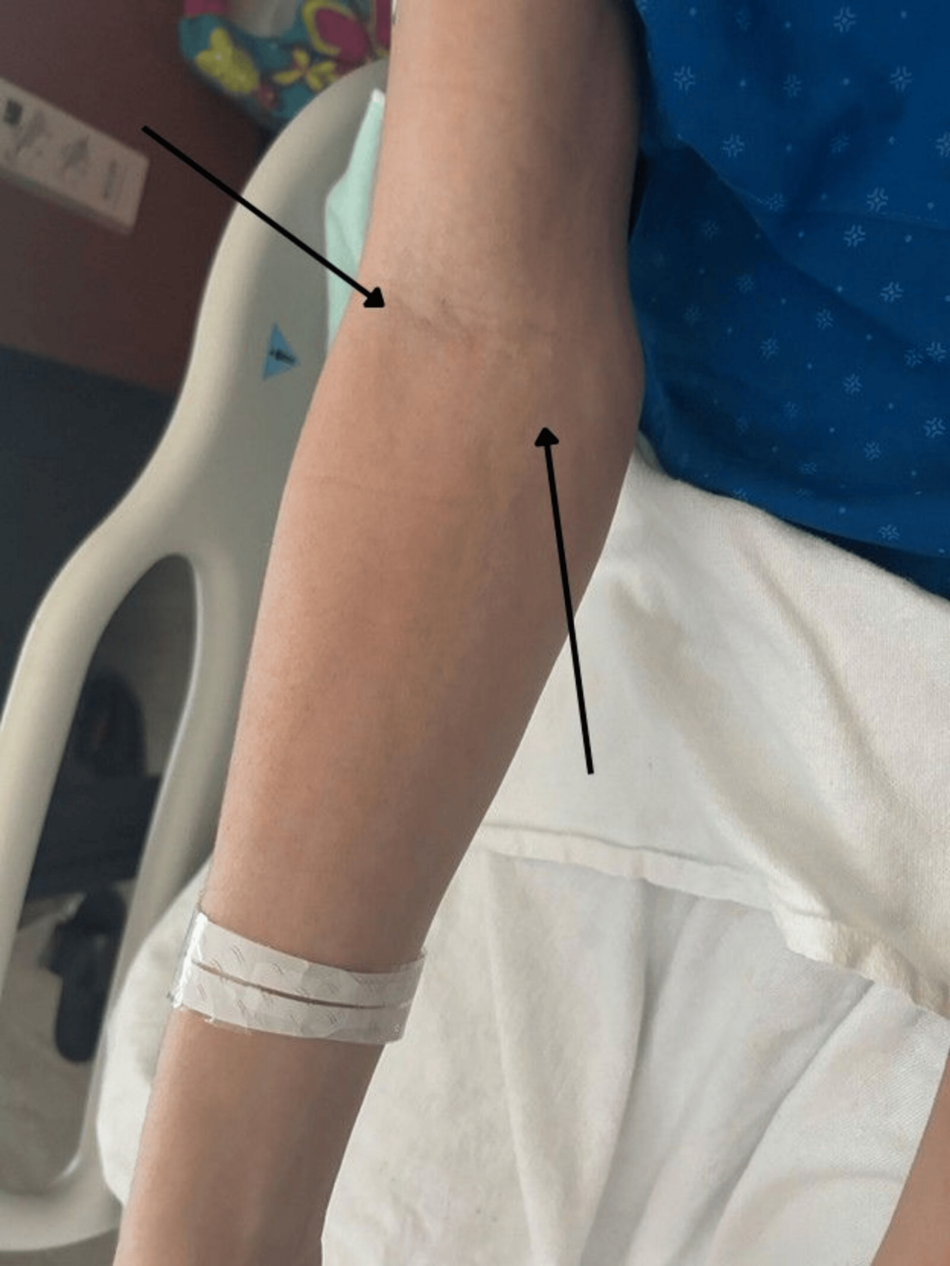
Clinical photograph of the left forearm showing skin tightening and induration consistent with eosinophilic fasciitis, with visible linear depressions along the course of superficial veins (groove sign), which is considered pathognomonic for eosinophilic fasciitis.

On physical examination, there was symmetrical induration and non-pitting edema of the forearms and legs, accompanied by tightening of the overlying skin, restricted hand mobility, and prominent superficial veins consistent with a positive groove sign (Figure 1). No rash, ulcers, or digital ischemia were observed.

Initial labs revealed microcytic, hypochromic anemia consistent with iron-deficiency anemia, elevated inflammatory markers, positive EBV IgG, and polymerase chain reaction (PCR) with negative IgM, suggesting prior infection with possible viral reactivation. Other autoimmune markers were negative (Table 1).

**Table 1:** Summary of laboratory findings MCV: mean corpuscular volume; WBC: white blood cell count; ESR: erythrocyte sedimentation rate; CRP: C-reactive protein; IGG: immunoglobulin g; IGA: immunoglobulin a; IGM: immunoglobulin m; IGE: immunoglobulin e; ANA: antinuclear antibody; RF: rheumatoid factor; ANTI-CCP: anti–cyclic citrullinated peptide antibody; SCL-70: anti–topoisomerase i antibody; EBV: Epstein–Barr virus; EBNA: Epstein–Barr nuclear antigen; CMV: cytomegalovirus; HIV: human immunodeficiency virus; PCR: polymerase chain reaction

Test/Category	Result	Reference Range	Units
Hemoglobin	9.1 (microcytic, hypochromic)	12–16 (female), 13–17 (male)	g/dL
MCV	78	80–100	fL
WBC	7.4	4–10.5	×10⁹/L
Platelets	250	150–450	×10⁹/L
ESR	101	<20	mm/hr
CRP	4.3	<10	mg/dL
Serum Iron	37	60–170	µg/dL
Iron saturation	14	20–50	%
Ferritin	143	30–400	ng/mL
EBV (EBNA IgG, IgM)	IgG positive, IgM negative	Negative	—
EBV DNA PCR	Positive	Negative	—
CMV, HIV, Lyme	Negative	Negative	—
ANA, RF, anti-CCP, Scl-70	Negative	Negative	—
IgG	4100	700-1600	mg/dL
IgA	704.7	70-400	mg/dL
IgM	345	40-230	mg/dL
IgE	306	<100	mg/dL
Kappa/Lambda ratio	0.92	0.26-1.65	—
Immunofixation	Polyclonal increase. No monoclonal spike detected	—	—

**Figure 2:**
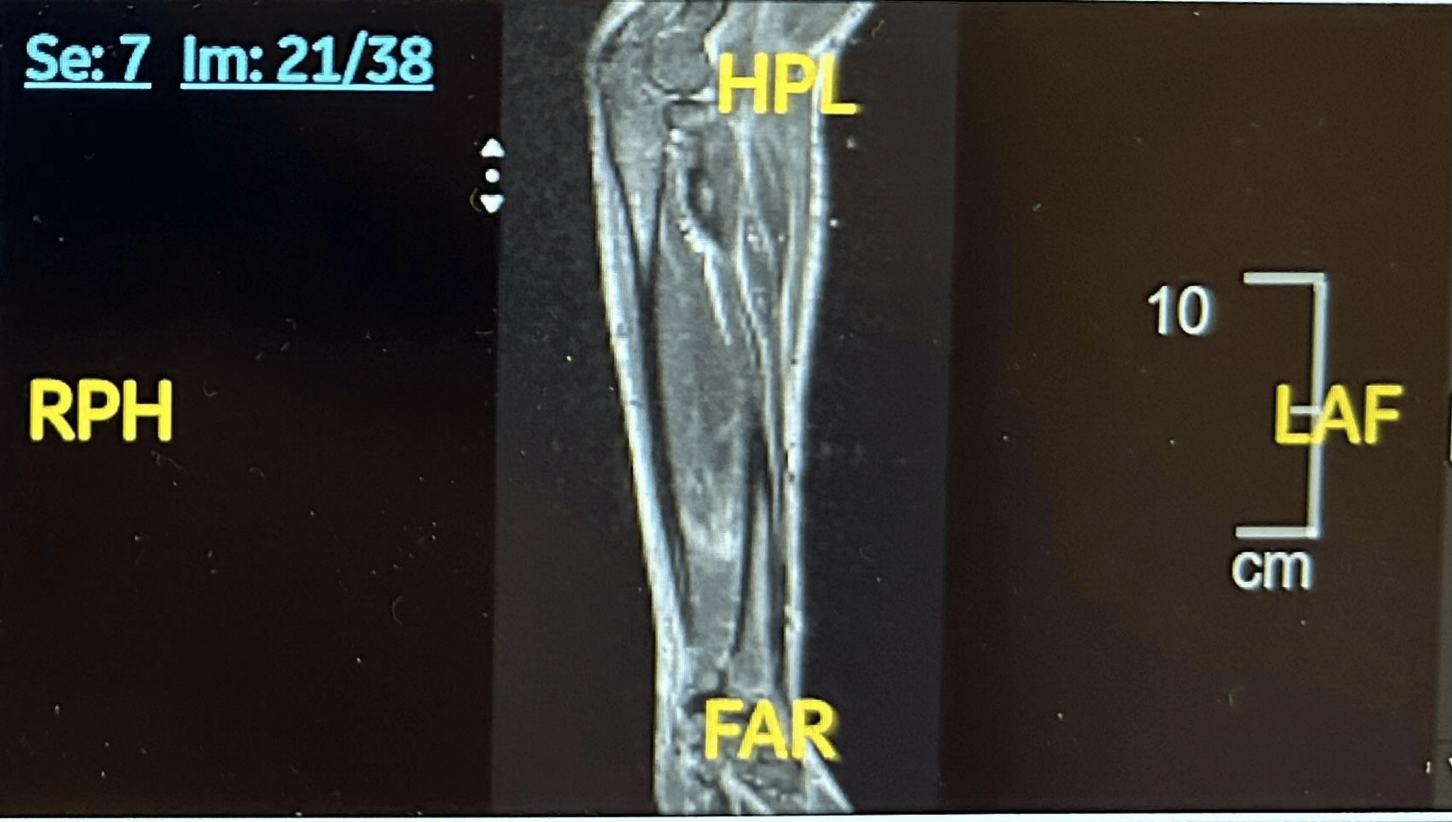
Coronal MRI of the right forearm demonstrates hyperintense signal along the deep fascial planes, consistent with diffuse fasciitis, particularly in the flexor compartment.

**Figure 3:**
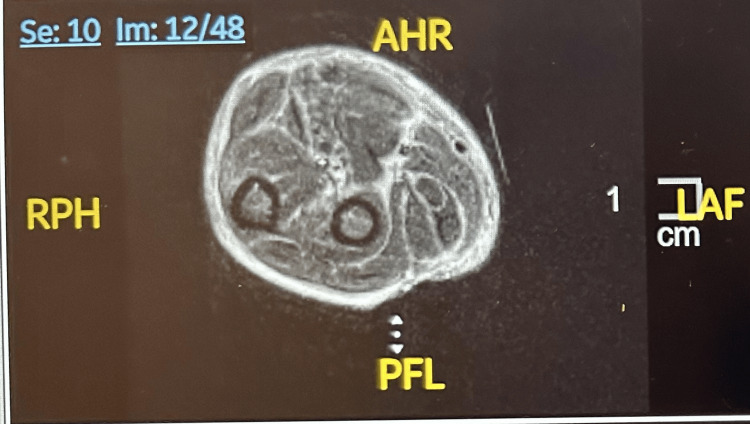
Axial MRI of the right forearm shows hyperintense signal along the deep fascial planes, consistent with diffuse fasciitis.

Imaging studies showed splenomegaly (14.6 cm) on computed tomography of the abdomen and pelvis. MRI of the forearms demonstrated diffuse fascial hyperintensity without fluid collections or necrosis (Figures 2, 3). A deep fascial biopsy showed chronic perivascular inflammation involving the subcutaneous tissue, fascia, dermis, and skeletal muscle, composed mainly of lymphocytes and plasma cells with scattered eosinophils, consistent with an inflammatory fasciitis pattern, and confirmed the diagnosis of EF.

The patient was treated with prednisone 60 mg daily, resulting in rapid improvement of stiffness and edema within 10 days. Fatigue improved with IV iron supplementation. He was discharged with outpatient rheumatology and hematology follow-up.

## Discussion

Diagnosing EF is often challenging because its clinical presentation overlaps with hematologic, infectious, and autoimmune conditions. Systemic manifestations such as anemia, splenomegaly, and hypergammaglobulinemia broaden the differential diagnosis [2,3]. In our case, negative serologies, immunofixation, imaging, and biopsy findings of no atypia or clonality helped exclude malignancy. The patient showed significant improvement with corticosteroid therapy, supporting a benign inflammatory process. The splenomegaly was likely secondary to EBV infection, which may have acted as an immunologic trigger for EF, later confirmed by biopsy. The role of EBV in EF remains speculative but is biologically plausible. EBV is known to persist in B cells and modulate immune function, predisposing to autoimmune or inflammatory responses [6]. Although adult cases of EF directly attributed to EBV are not well documented, infections are recognized triggers in EF, and mechanistic work shows that EBV can modulate innate and adaptive immunity in ways relevant to fibrosing autoimmunity [7]. In this patient, past EBV infection may have served as an immunologic trigger contributing to disease onset. It is important to involve a multidisciplinary team, including rheumatology, hematology, infectious disease, and pathology, to achieve an accurate diagnosis and provide effective treatment.

## Conclusions

EF is a rare and diagnostically complex condition that can mimic systemic autoimmune or hematologic disorders. In young patients with unexplained extremity swelling, stiffness, anemia, and splenomegaly, EF should be considered. This case also raises the possibility of EBV as a contributing factor in EF pathogenesis. Early recognition and corticosteroid therapy are essential for favorable outcomes.
